# Post-traumatic stress disorder and experiences involving violence or sexual abuse in a clinical sample of autistic adults with intellectual disabilities: Prevalence and clinical correlates

**DOI:** 10.1177/13623613231190948

**Published:** 2023-08-23

**Authors:** Arvid Nikolai Kildahl, Sissel Berge Helverschou

**Affiliations:** Oslo University Hospital, Norway

**Keywords:** autism spectrum disorders, intellectual disabilities, post-traumatic stress disorder, sexual abuse, trauma, violence

## Abstract

Autistic people appear to be at increased risk of post-traumatic stress disorder, and experiences involving violence or sexual abuse appear to be common in this population. However, knowledge concerning trauma in autistic people with co-occurring intellectual disabilities is limited. In this cross-sectional study, we explored the prevalence of post-traumatic stress disorder, as well as the prevalence and clinical correlates of reported experiences of violence and sexual abuse, in a clinical sample of 88 autistic adults with intellectual disabilities referred for mental health assessment. Only three participants (3.4%) were diagnosed with post-traumatic stress disorder, even if experiences involving violence (34.1%) or sexual abuse (17.0%) were common. Anxiety and affective disorders were common diagnoses among participants with these experiences. Controlling for autism characteristics, level of intellectual disability and communication skills, experiences of violence/sexual abuse were found to be associated with a measure of ‘challenging’ behaviours (Aberrant Behaviour Checklist). These results indicate that potentially traumatic experiences are common in autistic adults with intellectual disabilities referred for mental health assessment and that post-traumatic stress disorder may be under-recognised. The findings highlight the importance of trauma screening and post-traumatic stress disorder assessment, as well as the importance of trauma-informed care, in this population.

**Lay abstract:**

Post-traumatic stress disorder is a mental health disorder that may be triggered by the experience of events perceived as terrifying or overwhelming. Examples of such events include being the victim of violence or sexual abuse. Compared with other people, autistic people have increased risk of being exposed to violence or sexual abuse. In addition, autistic people may be more vulnerable to developing post-traumatic stress disorder following such exposure. However, knowledge is limited concerning the prevalence of experiences involving violence and sexual abuse, and post-traumatic stress disorder, in autistic people with co-occurring intellectual disabilities. Detection of these experiences, and identification of post-traumatic stress disorder, may be challenging in these individuals, and previous research indicates that post-traumatic stress disorder symptoms may be overlooked or misinterpreted. In this study, we examined prevalence of post-traumatic stress disorder, violence and sexual abuse in a clinical sample of 88 autistic adults with intellectual disabilities referred for mental health assessment. Only 3.4% were diagnosed with post-traumatic stress disorder, even if experiences involving violence (34.1%) or sexual abuse (17.0%) were common. Anxiety and affective disorders were common diagnoses among participants with these experiences. Controlling for autism characteristics, level of intellectual disability and communication skills, experiences of violence/sexual abuse were found to be associated with a measure of ‘challenging’ behaviours. These results indicate that potentially traumatic experiences are common in autistic adults with intellectual disabilities referred for mental health assessment, and that post-traumatic stress disorder may be under-recognised. The findings highlight the importance of trauma screening and post-traumatic stress disorder assessment, as well as the importance of trauma-informed care, in this population.

Post-traumatic stress disorder (PTSD) is a common sequela following experiences perceived as life-threatening or terrifying (American Psychiatric Association, 2013; Ehlers & Clark, 2000; van der Kolk, 2014). Symptoms include intrusions (flashbacks, intrusive memories), avoidance, negative alterations in mood and cognition, and alterations in arousal and reactivity (American Psychiatric Association, 2013). According to the *Diagnostic and Statistical Manual of Mental Disorders* (5th ed; *DSM*-5), PTSD is diagnosed following a predefined set of events, that is, when the individuals themselves have directly experienced, witnessed or learned that a close friend or relative has been exposed to death, severe injury, sexual violence or threats of these events (American Psychiatric Association, 2013).

## Prevalence of violence and sexual abuse in autistic people

Autistic people are at increased risk of experiencing violence (Hoover, 2015; Rumball, 2019). Compared with the controls, McDonnell et al. (2019) found significantly increased rates of physical abuse in autistic children, and autistic adults report higher rates of physical violence during childhood than non-autistic controls (Gibbs et al., 2021; Weiss & Fardella, 2018). Relying on caregiver report, Mandell et al. (2005) reported that 18.5% of autistic children had been physically abused. The increased risk of victimisation seems to continue into adulthood (Gibbs et al., 2022; Rumball, Brook, et al., 2021; Weiss & Fardella, 2018), with Gibbs and Pellicano (2023) reporting that violence was perceived as so common by autistic adults that such experiences had become normalised.

Autistic people, in particular autistic girls and women, are at increased risk of experiencing sexual abuse (Bargiela et al., 2016; Brown-Lavoie et al., 2014; Christoffersen, 2022; Dike et al., 2022; Gibbs et al., 2021; Gotby et al., 2018; Mandell et al., 2005; Pecora et al., 2019; Rumball, Brook, et al., 2021; Weiss & Fardella, 2018). In a survey study by Reuben et al. (2021), 72% of autistic adults reported having experienced sexual assault, unwanted/uncomfortable sexual experiences or physical assault, while Cazalis et al. (2022) reported 9/10 autistic women to have experienced sexual violence. A majority of these had experienced such events prior to age 15 years, and repeated victimisation was common.

As for autistic people with co-occurring intellectual disabilities, knowledge is more limited with regard to risk of violence and sexual abuse. Compared with the controls, McDonnell et al. (2019) found that autistic children with intellectual disabilities were more likely to experience physical and sexual abuse. In general, intellectual disability is associated with increased risk of experiencing violence and sexual abuse (Byrne, 2018), in children (Jones et al., 2012), adolescents (Reiter et al., 2007) and adults (Daveney et al., 2019; Fogden et al., 2016; Mevissen et al., 2016; Tomsa et al., 2021). Moreover, because autistic people with intellectual disabilities frequently depend on assistance from others in their daily lives, these individuals appear to be at risk of violence or injury occurring in the context of experiences that are uncommon or unusual in the general population. Such experiences include being coerced, inappropriate use of force, institutional abuse or negligence in services (Beadle-Brown et al., 2010; Codina et al., 2022; Daveney et al., 2019; Howlin & Clements, 1995; Kerns et al., 2022; Kildahl et al., 2020b; Kildahl & Jørstad, 2022; Strand et al., 2004).

## Prevalence of PTSD in autistic people

In a recent meta-analysis, Lai et al. (2019) found prevalence of PTSD in autistic people to vary from 0.3% to 6%. However, more recent findings have found probable PTSD in 32%–44% of smaller autistic samples (Haruvi-Lamdan et al., 2020; Reuben et al., 2021; Rumball, Brook, et al., 2021), suggesting that the prevalence of PTSD in autistic people has been underestimated. Specifically for autistic people with intellectual disabilities, Mehtar and Mukaddes (2011) found a 17.4% prevalence of PTSD in a clinical sample of autistic children/adolescents, of which 72.5% had a co-occurring intellectual disability. Other studies have failed to identify cases involving PTSD in autistic individuals with intellectual disabilities (de Bruin et al., 2007; Taylor & Gotham, 2016). Thus, the prevalence of PTSD in autistic individuals with intellectual disabilities remains unclear.

PTSD is caused by an interaction between the trigger, the potentially traumatic event and the specific individual’s vulnerabilities (Elwood et al., 2009; van der Kolk, 2014). Compared with non-autistic people, autistic people appear to have an increased vulnerability to PTSD following potentially traumatic events (Haruvi-Lamdan et al., 2020; Kerns et al., 2015; Rumball, 2019; Rumball, Brook, et al., 2021). Memory-related and cognitive processes that may differ for autistic people appear likely to be contributing to this vulnerability (Golan et al., 2022; Haruvi-Lamdan et al., 2018; Rumball, Antal, et al., 2021; Rumball, Brook, et al., 2021), including autobiographical memory (Coutelle et al., 2020, 2021; Crane et al., 2012) and perception of time (Vogel et al., 2019; see D’Argembeau, 2020), as well as brooding rumination (Golan et al., 2022), cognitive inflexibility (Golan et al., 2022) and thought suppression (Rumball, Antal, et al., 2021). Moreover, lack of appropriate coping strategies and social support (McCarthy et al., 2017; Mevissen et al., 2016), and lack of access to adapted assessment, treatment and care (Kerns et al., 2021; Stadnick et al., 2020) are likely to affect vulnerability to PTSD.

Vulnerability to PTSD may be further increased for autistic people with co-occurring intellectual disabilities. These individuals often have a more limited repertoire of coping strategies (Bakken et al., 2016; Kildahl et al., 2020b) and poorer access to social support (Mevissen et al., 2016). Their communication difficulties are often more extensive (Bakken et al., 2016), and it has been suggested that lack of access to conceptual, typically verbal language-based, processing strategies involves an increased risk of PTSD (Ehlers & Clark, 2000). In the general population, higher intelligence quotients (IQs) appear to be a protective factor for the development of PTSD (Breslau et al., 2006), and lower verbal IQs appear to be associated with increased risk of forming intrusive memories (Sündermann et al., 2013). Finally, these individuals’ reactions to traumatic experiences may be difficult for their families and caregivers to understand (Kildahl et al., 2020a, 2020b; Mitchell et al., 2021), involving a potential risk that these are met with inappropriate responses and therefore exacerbated.

## Challenges in assessment of PTSD in autistic people with intellectual disabilities

Mental health assessment in autistic people with intellectual disabilities often relies on information from proxy informants, due to these individuals’ often limited verbal communication abilities (Bakken et al., 2016; Deb et al., 2022; Kildahl et al., 2023). Thus, assessment of PTSD requires the traumatic experience to be known to, and understood as traumatic by, informants. Autistic people appear to be less likely to confide in others about such experiences (Gibbs et al., 2021), and individuals with co-occurring intellectual disabilities may struggle to communicate about traumatic experiences verbally (Kildahl et al., 2020b; Kildahl, Helverschou, & Oddli, 2020; Kildahl & Jørstad, 2022).

Recognition and identification of PTSD symptoms in autistic people with intellectual disabilities may be challenging (Kerns et al., 2023; Kildahl et al., 2019, 2020b; Rittmannsberger et al., 2019). PTSD symptoms may be misattributed to anxiety or depressive disorders, as the most easily observable behavioural manifestations of PTSD in this population appear to overlap with behavioural manifestations of these disorders (Kildahl et al., 2019, 2020a, 2020b; Kildahl, Helverschou, & Oddli, 2020). Moreover, while anxiety and depressive disorders are diagnosed from symptom manifestations alone, diagnosing PTSD requires substantiating an association between a previous traumatic experience and the development of symptoms, in addition to recognising the appropriate symptoms (American Psychiatric Association, 2013). Thus, diagnosing PTSD in autistic people with intellectual disabilities will require practitioners to make more extensive inferences about these individuals’ subjective experience than diagnosing anxiety or depression (Kildahl et al., 2021, 2023).

PTSD symptoms may also be misattributed to the individual’s autism (Brenner et al., 2018; Kildahl et al., 2020b; Kildahl, Helverschou, & Oddli, 2020) or intellectual disability (Kildahl et al., 2023; Mevissen et al., 2016), often referred to as diagnostic overshadowing (Jopp & Keys, 2001; Reiss et al., 1982). In addition, autism and intellectual disability are heterogeneous conditions (Burack et al., 2021; Lai et al., 2014). In order to avoid diagnostic overshadowing, it is therefore likely to be necessary to explore and interpret the individual’s symptoms and behaviours in context of their specific situation and development (Deb et al., 2022; Kerns et al., 2023; Kildahl et al., 2023).

Previous studies have found associations between trauma and ‘challenging’ behaviours in people with intellectual disabilities (McDonnell et al., 2019; McNally et al., 2021; Rittmannsberger et al., 2020), including autistic individuals (Kerns et al., 2023; Kildahl, Oddli, & Helverschou, 2020; McDonnell et al., 2019; Mehtar & Mukaddes, 2011), indicating that ‘challenging’ behaviours may constitute a behavioural manifestation of PTSD symptoms. Four studies have used the Aberrant Behaviour Checklist (ABC; Aman, 2012; Aman et al., 1985), an instrument with scales measuring five different domains of ‘challenging’ behaviours: In a sample of adults with intellectual disabilities, Rittma-nnsberger et al. (2020) found associations between trauma exposure and three scales (irritability/agitation/crying, hyperactivity/noncompliance, inappropriate speech), which was mediated by PTSD symptoms. Brenner et al. (2018) found higher scores on two scales (irritability/agitation/crying, lethargy/social withdrawal) in autistic children with reported abuse experience, while Mehtar and Mukaddes (2011) identified an association between trauma and a subscale including some items from irritability/agitation/crying. In autistic adults with intellectual disabilities, Kildahl, Oddli, and Helverschou (2020) identified associations between serious disease/injury in a close relative/caregiver/friend and higher scores on irritability/agitation/crying and hyperactivity/noncompliance.

## The current study

In the current study, we aim to explore the prevalence of PTSD diagnoses and two reported traumatic experiences meeting the formal criteria for PTSD, in a clinical sample of autistic adults with intellectual disabilities referred for mental health assessment. We chose to focus specifically on whether participants themselves had been exposed to violence and sexual abuse. These events are of a severity that makes them likely to be reported if proxy informants are aware of them. Moreover, direct exposure to such events may be more likely to be known by proxy informants than more indirect exposure, such as witnessing others experiencing these events. In addition, we aim to explore associations between these experiences and clinical/behavioural characteristics as reported on two standardised checklists (ABC and Psychopathology in Autism Checklist (PAC)), while controlling for autism characteristics, level of intellectual disability and communication skills.

## Method

### Design and setting

This study uses data from the AUP (Autism, Intellectual Disabilities, Mental Illness) study (Helverschou, Bakken, et al., 2021), a longitudinal study involving eight centres throughout Norway providing specialist, hospital-level mental health services for autistic people with intellectual disabilities. Data were collected from 2010 to 2020. The current study uses data from one of these centres, a specialist mental health department for people with intellectual disabilities accepting referrals only from other hospital-level services. The department provides inpatient and outpatient services across a large geographical area. Interdisciplinary assessments are typically conducted over several months and involve psychologists, psychiatrists, and mental health or intellectual disability nurses. All referrals to the centre are due to suspicion of a co-occurring mental health disorder, and cases are generally complex. The current study uses data from T1, collected at referral/start of the assessment, and from T2, data collected following treatment, typically a year after T1.

### Participants

Prior to inclusion, all participants in the AUP study had been assessed for, and diagnosed with, co-occurring autism spectrum disorder and intellectual disability according to the International Classification of Diseases (ICD)-10 (World Health Organization, 1992). No exclusion criteria were applied with regard to further co-occurring disabilities or somatic diagnoses. For the current study, all participants from the centre in question with complete or near-complete (missing <4 items on the two checklists) data sets for the relevant measures were included.

The current study included 88 participants, aged 15–68 years (*M* = 27.58, *SD* = 10.82), 26 (29.5%) female and 62 (70.5%) male. A total of 73 (83%) were diagnosed with mild or moderate intellectual disabilities (27.4% female), while 15 (17%) were diagnosed with severe or profound intellectual disability (40% female). Following assessment, 83 participants (94.3%) were diagnosed with at least one mental health disorder. These included anxiety disorders (37.5%), affective disorders (35.2%), psychotic disorders (30.7%), obsessive-compulsive disorders (OCD; 10.2%), personality disorders (6.8%) and other diagnoses (6.8%). Most participants were diagnosed with one mental health disorder (65.9%), while 27.3% were diagnosed with two and 2.3% with three.

Specific data on race/ethnicity and socioeconomic status were not recorded.

### Research ethics

The AUP study was approved by the Privacy Data Protection Supervisor (Local institutional review board), Oslo University Hospital (#2010/19579). Owing to participants’ limited capacity to consent, written consent was obtained for all participants from their legal guardians. In addition, participants consented themselves when this was feasible.

### Materials

#### PTSD diagnoses

All final diagnoses included for the current study were the result of comprehensive and interdisciplinary assessments based on the AUP protocol, usually with use of additional assessment tools and clinical observation (Helverschou, Bakken, et al., 2021). As mental health assessment in autistic people with intellectual disabilities frequently takes time (Bakken et al., 2016; Dalhaug et al., 2023; Underwood et al., 2010, 2015), mental health diagnoses were reported by clinicians (psychiatrist or clinical psychologist) at T2 of the study. Final diagnostic conclusions for all participants in the current study were discussed in interdisciplinary meetings. The AUP study did not specifically screen for PTSD, and the final diagnoses reported are those following a comprehensive assessment in standard clinical practice in a specialised mental health department.

#### Trauma experiences

Trauma experiences according to PTSD criteria were reported by clinicians in the AUP study, on a form including numerous psychiatric symptoms (Helverschou, Bakken, et al., 2021). In addition, patient charts were checked for information about experiences of violence or sexual abuse. These experiences were coded as dichotomous variables (*no* = 0, *yes* = 1). A code of ‘yes’ was chosen if the presence of such experiences was confirmed, or a strong suspicion involving a specified scenario had been reported and noted in the patient’s chart (e.g. a suspicion involving a specific perpetrator, location, and time). Non-specific suspicions were coded as ‘no’.

#### Psychopathology in Autism Checklist

The PAC (Helverschou et al., 2008, 2009) is an informant-completed mental health screening tool developed for autistic individuals with intellectual disabilities. It was constructed by identifying mental health disorder symptom descriptions and diagnostic criteria judged by a panel of expert clinicians not to overlap with autism-related behaviours (Helverschou et al., 2008). The psychometric properties of the PAC have been found to vary from acceptable to good (Helverschou et al., 2008, 2009; Helverschou, Ludvigsen, et al., 2021), and appears to distinguish reliably between autistic adults with intellectual disabilities according to whether they have a co-occurring mental health disorder or not, and to some extent between different mental health disorders (Dalhaug et al., 2023; Helverschou et al., 2008, 2009; Helverschou, Ludvigsen, et al., 2021).

The PAC comprises 42 items rated in two domains: ‘Extent of problems’ (*not a problem* = 1, *minor problem* = 2, *moderate problem* = 3, *severe problem* = 4) and ‘Change from usual behaviour’ (worsened, unchanged, improved). Scores are distributed across five scales: psychosis (10 items), depression (7 items), anxiety disorders (6 items), obsessive-compulsive disorder (OCD; 7 items) and general adjustment problems (12 items), and cut-off values for screening exist for all scales (Helverschou et al., 2009). In addition to use of these cut-offs, PAC scores were used as dimensional measures, reflecting the severity of the reported symptoms within these domains. When the very few missing values were treated as *not a problem*, overall internal consistency of the PAC was good in this sample (Cronbach’s α = 0.90), as well as good to acceptable for the scales for psychosis (α = 0.79), depression (α = 0.79), anxiety disorders (α = 0.69), obsessive-compulsive disorders (α = 0.78) and general adjustment difficulties (α = 0.71). Depending on the participant’s living arrangement, informants completing the PAC were either professional caregivers or family members.

#### Aberrant Behaviour Checklist

Developed to evaluate treatment responses in people with intellectual disabilities (Aman, 2012; Aman et al., 1985), the ABC is an informant-completed checklist for ‘challenging’ behaviours (Aman, 2012; Halvorsen et al., 2022; Helverschou et al., 2020). It is a widely used measure and good psychometric properties have been demonstrated across varying levels of intellectual disabilities (Aman, 2012; Flynn et al., 2017), in autistic children/adolescents (Brinkley et al., 2007; Kaat et al., 2014), and for its Norwegian version (Halvorsen et al., 2019). The ABC comprises 58 items rated on a four-point scale (*not a problem* = 0, *minor problem* = 1, *moderate problem* = 2, *severe problem* = 3). Scores are distributed across five scales: irritability/agitation/crying (15 items), lethargy/social withdrawal (16 items), stereotypic behaviour (7 items), hyperactivity/noncompliance (16 items) and inappropriate speech (4 items). The ABC is a dimensional measure, and higher scores on the respective scales reflect more severe levels of challenging behaviour within these domains. When the very few missing items were treated as *not a problem*, internal consistency of the ABC was good in this sample (Cronbach’s α = 0.94), as well as for the scales irritability/agitation/crying (α = 0.92), lethargy/social withdrawal (α = 0.87), stereotypic behaviour (α = 0.85), hyperactivity/noncompliance (α = 0.91) and inappropriate speech (α = 0.79). Depending on the participant’s living arrangement, informants completing the ABC were either professional caregivers or family members.

#### Social Communication Questionnaire

Autism-related characteristics were measured using the Social Communication Questionnaire (SCQ; Rutter et al., 2003), current version. The SCQ contains 40 yes/no items describing such ‘symptoms’ and was completed by informants (professional caregivers or family members). Because ranges for SCQ scores differ by level of verbal language skills, scores were recalculated to make ranges 0–39 for all participants.

#### Vineland Adaptive Behaviour Scales-Communication

Participants’ communication skills were measured using standard scores from the Vineland Adaptive Behaviour Scales (VABS), second edition, expanded version, communication scale (Sparrow et al., 2008).

#### Level of intellectual disability

Level of intellectual disability was reported by clinicians in the AUP study as a dichotomous variable, mild/moderate (score = 0) or severe/profound (score = 1), based on a clinical judgement informed by previous diagnoses, clinical background information from hospital records, observation by expert clinicians and scores from the VABS, second edition, expanded version (Sparrow et al., 2008). More comprehensive data regarding intellectual abilities were not available due to the difficulties of obtaining reliable IQ results from patients with severe cognitive, behavioural and/or mental health difficulties.

### Procedure

Data in the AUP study were collected as a part of the regular clinical service provision according to a prespecified protocol (Helverschou, Bakken, et al., 2021). All data for the current study were collected at T1 (after referral), except for information about mental health disorder diagnoses (reported at T2), and information about trauma experiences (reported at T2 and checked in patient charts). The same informant(s) usually completed the ABC, PAC and SCQ at T1.

### Analysis

Frequency analyses were conducted for PTSD, violence and sexual abuse, as well as for final mental health diagnoses for participants having these experiences. An alpha value of 0.05 was chosen for the statistical analyses, due to the exploratory nature of the study. Binary logistic regressions were used to explore whether the likelihood of having experienced violence or sexual abuse differed according to gender or level of intellectual disability. Mann–Whitney U tests were used to check whether participants having experienced violence or sexual abuse differed with regard to age and SCQ/VABS-C scores.

The very few missing items on PAC/ABC were treated as *not a problem*. Frequency analyses were conducted for PAC scores over cut-off among participants having experienced violence or sexual abuse. Binary logistic regressions were used to check whether these participants differed with regard to the likelihood of scoring over PAC cut-offs.

Group differences between participants having experienced either violence or sexual abuse and the remaining sample were explored for PAC and ABC scales using a nonparametric test, the Mann–Whitney U test.

Further exploration of associations with PAC and ABC scales were investigated using hierarchical linear regression (ENTER model). Hierarchical linear regression is used for model comparison, allowing for comparisons of the explained variance in a dependent variable according to what predictors are entered into the model. ABC and PAC scales were the dependent variables for the regressions. Because age, gender, level of intellectual disability, verbal language skills and autism-related characteristics may affect the scores on these scales (e.g. Kildahl, Oddli, & Helverschou, 2020; Painter et al., 2018), and affect the behavioural manifestations of trauma-related symptoms (Kildahl et al., 2020b), these were entered in Block 1. Violence and sexual abuse were entered in Block 2.

The Benjamini–Hochberg procedure (Benjamini & Hochberg, 1995) was used to control for multiple comparisons, using a false discovery rate of 0.05.

### Community involvement

A.N.K. is a clinical psychologist working with autistic people with intellectual disabilities and their families. There was no other community involvement for the current study.

## Results

### Prevalence of PTSD, violence and sexual abuse

Only three participants (3.4%) had been diagnosed with PTSD, two females and one male, all diagnosed with severe/profound intellectual disabilities. In accordance with the diagnostic criteria for PTSD, one had experienced violence, one had experienced sexual abuse and one had experienced both the events.

Violence was reported for 30 (34.1%) participants, 23 males (37.1%) and 7 females (26.9%). Experiences included severe institutional abuse, caregiver or domestic violence, and random assaults. For participants with mild/moderate intellectual disabilities, 23 participants (31.5%) had experienced violence, while 7 (46.7%) of the participants with severe/profound intellectual disabilities had these experiences. No significant effects of gender or level of intellectual disability were found for violence using binary logistic regression. Using the Mann–Whitney U test, no significant differences were found for age, SCQ or VABS-C, according to whether or not participants had experienced violence.

Sexual abuse was reported for 15 (17.0%) participants, 5 females (19.2%) and 10 males (16.1%). Experiences included repeated sexual abuse over time by family members, service providers or peers, as well as single incidents of sexual assault. Sexual abuse was reported in 12 (16.4%) participants with mild/moderate intellectual disabilities, and in 3 (20.0%) participants with severe/profound intellectual disabilities. No significant effects of gender or level of intellectual disability were found for sexual abuse using binary logistic regression. Using the Mann–Whitney U test, no significant differences were found for age, SCQ or VABS-C, according to whether or not participants had experienced sexual abuse.

For 6 participants (6.8%; 3 females, 3 males; 5 mild/moderate, 1 severe/profound) co-occurring violence and sexual abuse was reported. In other words, 40% of the participants who had experienced sexual abuse had also experienced violence. In total, almost half the sample (39 participants, 44.3%) had experienced at least one of these events, of which only 3 (7.7%) were diagnosed with PTSD.

### Violence, sexual abuse and mental health disorder diagnoses

Mental health diagnoses for the 30 participants having experienced violence included anxiety disorders (14, 46.7%), affective disorders (10, 33.3%), psychotic disorders (9, 30.0%), depressive disorders (5, 16.7%), personality disorders (3, 10%), OCD (2, 6.7%) and PTSD (2, 6.7%).

For sexual abuse, diagnoses for the 15 affected participants included affective disorders (7, 46.7%), anxiety disorders (6, 40.0%), personality disorders (4, 26.7%), PTSD (2, 13.3%) and psychotic disorders (1, 6.7%).

### Violence, sexual abuse and behavioural measures (PAC/ABC)

#### PAC scores over cut-off

Of the 30 participants having experienced violence, 24 (80.0%) scored over the cut-off on PAC general adjustment difficulties, 22 (73.3%) on PAC anxiety, 20 (66.7%) on PAC depression, 17 (56.7%) on PAC psychosis and 9 (30.0%) on PAC OCD. Binary logistic regressions revealed that participants having experienced violence were more likely than those who had not to score over the cut-off for PAC anxiety (*OR* = 2.95, 95% CI [1.13, 7.69], *p* = 0.027), and PAC psychosis (*OR* = 2.49, 95% CI [1.01, 6.13], *p* = 0.027). No other significant effects of violence were found for PAC scales over cut-off.

Of the 15 participants having experienced sexual abuse 12 (80%) scored over the cut-off on PAC general adjustment difficulties, 9 (60.0%) on PAC depression, 8 (53.3%) on PAC anxiety, 4 (26.7%) on PAC psychosis and 2 (13.3%) on PAC OCD. Binary logistic regressions revealed no significant effects of sexual abuse for whether PAC scores were over cut-off.

#### Group differences for violence and sexual abuse

Using PAC/ABC continuous scores, Mann–Whitney U tests for group differences revealed significant differences between participants who had experienced violence, and those who had not, for PAC general adjustment difficulties, PAC psychosis, PAC anxiety, ABC irritability/agitation/crying, ABC stereotypical behaviour, ABC hyperactivity/noncompliance and ABC inappropriate speech; see Table 1. Effect sizes were small.

**Table 1.** Group differences for PAC/ABC scores according to whether participants had experienced violence, using Mann–Whitney U tests.

	Min–max	Total sample			Experience of violence reported		*U*	*z*	*p*	*r*
					Yes (*n* = 30)	No (*n* = 58)				
		*Range*	*M*	*SD*	*Mdn*	*Mdn*				
PAC general adjustment difficulties	12–48	14–42	28.75	6.50	33	28	622.50	−2.18	0.029*	–0.11
PAC psychosis	10–40	10–36	21.33	6.37	23.5	20	596.00	−2.50	0.012*	–0.13
PAC depression	7–28	8–28	15.98	5.02	16.5	15	705.00	−1.46	0.145	–0.07
PAC anxiety	6–24	6–21	11.75	3.89	12	10	614.50	−2.26	0.024*	–0.11
PAC obsessive-compulsive disorder	7–28	7–27	13.25	4.96	13.5	11	682.50	−1.66	0.098	–0.08
ABC irritability/agitation/crying	0–45	0–43	17.17	11.56	23	11.5	533.00	−2.97	0.003**	–0.15
ABC lethargy/social withdrawal	0–48	1–38	15.40	9.46	15	12.5	835.00	−0.31	0.758	–0.02
ABC stereotypic behaviour	0–21	0–17	5.53	4.90	6	4	643.00	−2.01	0.045*	–0.10
ABC hyperactivity/noncompliance	0–48	0–46	17.42	11.28	23.5	13	562.00	−2.71	0.007**	–0.14
ABC inappropriate speech	0–12	0–12	4.50	3.69	6	3	623.50	−2.18	0.029*	–0.11

PAC: Psychopathology in Autism Checklist; ABC: Aberrant Behaviour Checklist; *SD*: standard deviation.

* *p* ⩽ 0.05; **p ⩽ 0.01.

Mann–Whitney U tests for group differences revealed no significant differences between participants who had experienced sexual abuse and those who had not.

#### Hierarchical regressions for PAC/ABC scales

In the hierarchical regressions controlling for age, gender, SCQ, VABS-C and level of intellectual disability, adding violence and sexual abuse to the model did not significantly increase the explained variance for any PAC scales.

Using the same analysis for ABC scales, adding violence and sexual abuse to the model significantly increased the explained variance for ABC irritability/agitation/crying (*ΔR*^2^ = 0.117, *F*(2, 77) = 6.47, *p* = 0.003) and ABC hyperactivity/noncompliance (*ΔR*^2^ = 0.101, *F*(2, 77) = 5.42, *p* = 0.006); see Table 2. Adding violence and sexual abuse to the model did not significantly increase the explained variance for the remaining ABC scales.

**Table 2.** Hierarchical multiple regression for violence and sexual abuse on ABC irritability/agitation/crying and ABC hyperactivity/noncompliance.

Block	Predictors	Unstandardized coefficients			Standardised coefficients		*R* ^2^	*Adjusted R* ^2^	*ΔR* ^2^	*F*	*p*
		*B*	*SE*	95% CI	*ß*	*p*					
ABC irritability/agitation/crying											
1							0.185	0.133	0.185	3.58	0.006**
	Age	−0.09	0.12	[−0.32, 0.15]	−0.08	0.462					
	Gender	−4.50	2.64	[−9.75, 0.74]	−0.18	0.092					
	SCQ	0.43	0.20	[0.04, 0.83]	0.28	0.031*					
	VABS–C	−0.09	0.10	[−0.29, 0.12]	−0.13	0.387					
	Level of intellectual disability	2.76	4.03	[−5.26, 10.77]	0.09	0.684					
2							0.302	0.238	0.117	4.76	<0.001***
	Age	−0.14	0.11	[−0.36, 0.09]	−0.13	0.222					
	Gender	−5.25	2.49	[−10.20, −0.30]	−0.21	0.038*					
	SCQ	0.55	0.19	[0.17, 0.93]	0.35	0.005**					
	VABS–C	−0.03	0.10	[−0.23, 0.17]	−0.04	0.756					
	Level of intellectual disability	3.06	3.78	[−4.46, 10.58]	0.10	0.420					
	**Violence**	**6.39**	**2.39**	**[1.62, 11.15]**	**0.26**	**0.009** **					
	**Sexual abuse**	**6.87**	**3.03**	**[0.84, 12.90]**	**0.23**	**0.026** *					
ABC hyperactivity/noncompliance											
1							0.183	0.131	0.183	3.53	0.006**
	Age	−0.31	0.12	[−0.54, −0.09]	−0.30	0.008**					
	Gender	0.63	2.57	[−4.49, 5.75]	0.03	0.806					
	SCQ	0.25	0.19	[−0.13, 0.63]	0.17	0.193					
	VABS–C	−0.08	0.10	[−0.28, 0.12]	−0.12	0.436					
	Level of intellectual disability	5.58	3.93	[−2.24, 13.41]	0.19	0.159					
2							0.283	0.218	0.101	4.35	<0.001***
	Age	−0.36	0.11	[−0.58, −0.14]	−0.34	0.002**					
	Gender	−0.07	2.46	[−4.96, 4.82]	−0.00	0.978					
	SCQ	0.35	0.19	[−0.02, 0.73]	0.23	0.064					
	VABS–C	−0.03	0.10	[−0.22, 0.17]	−0.04	0.800					
	Level of intellectual disability	5.86	3.73	[−1.56, 13.29]	0.20	0.120					
	**Violence**	**6.02**	**2.36**	**[1.31, 10.72]**	**0.25**	**0.013** *					
	**Sexual abuse**	**5.83**	**2.99**	**[−0.13, 11.79]**	**0.20**	**0.055**					

ABC: Aberrant Behaviour Checklist; *SE*: standard error; CI: confidence interval; SCQ: Social Communication Questionnaire; VABS-C: Vineland Adaptive Behaviour Scales-Communication.

* *p* ⩽ 0.05; **p ⩽ 0.01; ***p ⩽ 0.001. The values in bold are concerning the variables of interest in this study.

### Control for multiple comparisons

Application of the Benjamini–Hochberg procedure for correction of multiple comparisons, using a false discovery rate of 0.05, showed that only the *p*-values for the regression models including violence and sexual abuse for ABC irritability/agitation/crying and ABC hyperactivity/noncompliance were robust.

## Discussion

In a clinical sample of autistic adults with intellectual disabilities referred for mental health assessment, a substantial proportion had previous experiences involving violence or sexual abuse. However, while traumatic experience does not necessarily result in development of PTSD, PTSD diagnoses were rare, even if the clinicians making mental health disorder diagnoses had reported these experiences in the participants’ charts. For individuals having experienced violence or sexual abuse, diagnoses of anxiety or depressive disorders were common. Experiences involving violence and sexual abuse were associated with a measure of ‘challenging’ behaviour previously found to be associated with trauma and PTSD. These findings indicate that PTSD may have been an underutilised diagnosis in this sample, in line with previous suggestions that PTSD is under-recognised in autistic adults with intellectual disabilities.

Exposure to violence and sexual abuse was common in this clinical sample, with almost half the participants having experienced at least one of these events. These results are in line with previous findings indicating that trauma exposure is common in autistic people (Gibbs et al., 2021; Haruvi-Lamdan et al., 2020; Reuben et al., 2021; Rumball, 2019; Rumball et al., 2020), and people with intellectual disabilities (Daveney et al., 2019; Mevissen et al., 2016; Tomsa et al., 2021; Wigham & Emerson, 2015). Comparisons with general population samples are challenging, because there are considerable differences between males and females regarding risk of violence or sexual abuse (Dale et al., 2023; Hébert et al., 2019; Pereda et al., 2009), and the current sample included few females and were a highly selected clinical sample. However, the prevalence of sexual abuse in the current sample appear to be higher than findings from general population studies (e.g. Dale et al., 2023; Hébert et al., 2019; Pereda et al., 2009), while the prevalence of violence appear to be comparable to findings from the general population (e.g. Dale et al., 2023). However, these comparisons need to be interpreted with caution because data from the general population are usually based on self-report, while the current data was based on informant report and violence or sexual transgressions may not have been known to, and consequently not reported by, informants (Kildahl, Helverschou, & Oddli, 2020).

The most easily observable PTSD symptoms in autistic people with intellectual disabilities appear to overlap with behavioural manifestations of anxiety and depression (Kildahl et al., 2020a). While only three participants had been diagnosed with PTSD, diagnoses of anxiety or depressive disorder were frequent among participants exposed to violence or sexual abuse. Rumball, Antal, et al. (2021) report similar results from a sample of autistic people without intellectual disabilities, where a majority of participants were diagnosed with anxiety disorders, and not PTSD, despite previous traumatic experience. Thus, the current results indicate that PTSD was an underutilised diagnosis in this sample, and PTSD symptoms may have been misinterpreted as anxiety/depressive symptoms. In spite of this, no associations were found for these experiences with PAC anxiety or PAC depression. This may be due to limitations of the PAC, which is a screening tool and not a diagnostic tool (Helverschou et al., 2009; Helverschou, Ludvigsen, et al., 2021). Moreover, more than 90% of the current sample were diagnosed with a mental health disorder, indicating that a possible reason no specific associations were found for PAC anxiety/depression was that these symptoms were common in the sample. For instance, 30.7% were diagnosed with psychotic disorders, in which anxiety and depressive symptoms appear to be common in this specific population (Bakken et al., 2023).

Somewhat surprisingly, the participants diagnosed with PTSD all had severe/profound intellectual disabilities. The reasons for this are unclear, but it is possible that these participants had particularly extensive and severe trauma histories, and that behavioural manifestations of PTSD symptoms were clearly observable due to the severity of symptoms (Kildahl et al., 2020a).

Previous studies (e.g. Kerns et al., 2023; Kildahl et al., 2019; Kildahl, Helverschou, & Oddli, 2020) have indicated a risk that PTSD symptoms are misattributed to the individual’s autism-related characteristics, indicating a risk of diagnostic overshadowing. PTSD and anxiety disorders appear to be highly prevalent in autistic people (Haruvi-Lamdan et al., 2020; Hollocks et al., 2019; Kerns et al., 2021; Lai et al., 2019; Reuben et al., 2021; Rumball, Brook, et al., 2021). In the current study, almost half the participants had experienced at least one event within the diagnostic criteria for PTSD. It is possible that mental health professionals working with autistic people have become so accustomed to observing stress, anxiety and trauma in autistic people that these symptoms are at risk of being overlooked, because they occur in almost all autistic individuals referred for mental health services. This indicates that specific exploration of potential trauma and PTSD symptoms in autistic people with intellectual disabilities may be necessary to recognise these (Kerns et al., 2023; Kildahl et al., 2020b). Although their psychometric properties are unclear in autistic people, trauma-specific measures developed for people with intellectual disabilities such as the Lancaster and Northgate Trauma Scale – Intellectual Disabilities (Wigham et al., 2011) and the Trauma Information Form (Hall et al., 2014) may aid in such exploration, along with obtaining a clinical trauma history (Kildahl & Jørstad, 2022).

The only statistically robust clinical associations identified for violence and sexual abuse in the current sample were two ABC scales, measuring ‘challenging’ behaviours. These results are line with previous studies exploring associations between this measure and trauma/PTSD (Brenner et al., 2018; Kildahl, Oddli, & Helverschou, 2020; Mehtar & Mukaddes, 2011; Rittmannsberger et al., 2020). Kildahl, Oddli, and Helverschou (2020) have previously pointed out that some items on these ABC scales (irritability/agitation/crying, hyperactivity/noncompliance) overlap with PTSD symptom manifestations described in autistic people with intellectual disabilities. Such items include irritability, anger outbursts and self-destructive behaviours, behaviours that are frequently described as ‘disruptive’ or ‘challenging’ when occurring in this population. Irritability is included in the diagnostic criteria for PTSD as a symptom of altered arousal and reactivity (American Psychiatric Association, 2013). Unless the individual’s altered arousal and reactivity is considered in their day-to-day services, it is perhaps not surprising that irritability in people with limited verbal communication skills may result in behaviours or coping strategies perceived by others as ‘challenging’. Similarly, avoidance may easily be perceived as ‘noncompliance’ if most of the individual’s tasks are performed at the instruction of caregivers. While one previous finding indicates that the association between trauma and ‘challenging’ behaviours is mediated by severity of PTSD symptoms (Rittmannsberger et al., 2020), the relationship between trauma and ‘challenging’ behaviour is likely to be bidirectional. For example, people displaying ‘challenging’ behaviours may be at increased risk of trauma from inappropriate management of these behaviours (Daveney et al., 2019; Kildahl & Jørstad, 2022; Kildahl et al., 2020b; Strand et al., 2004).

Adequate recognition of trauma and PTSD, including identification and management of potential triggers, may be critical for appropriate adaptation of day-to-day services for autistic people with intellectual disabilities (Kildahl et al., 2021; Kildahl & Jørstad, 2022; Rich et al., 2021; Truesdale et al., 2019). Trauma-informed care is a systems-focused approach focusing on safety, trustworthiness, choice, collaboration and empowerment (Keesler, 2014). This approach has been held as critical in future service development for people with intellectual disabilities (Truesdale et al., 2019). The prevalence of potentially traumatic experiences found in the current study underlines the importance of this approach in services for autistic people with intellectual disabilities, including for individuals not necessarily diagnosed with PTSD. Finally, the mechanisms in the apparent underutilisation of PTSD diagnoses in this population is an important topic for future studies.

### Limitations

The current study was based on cross-sectional data, and no causal interpretations are possible. The study was based on a small clinical sample, with most participants being diagnosed with co-occurring mental health disorder. Therefore, these findings are not generalizable to non-clinical samples. Furthermore, the study was conducted using an existing data set, and this data set did not include a measure of PTSD, making it unclear how many of the participants met criteria for this diagnosis. The lack of a PTSD-specific measure also limits further disentanglement of the relationship between PTSD and ‘challenging’ behaviours. The lack of further associations for violence and sexual abuse on PAC/ABC may be due to the study being insufficiently powered to detect an effect, in particular for sexual abuse.

Because this study focused specifically on violence and sexual abuse, other potentially traumatic experiences (including experiences not formally qualifying for PTSD in the *DSM*-5; Kerns et al., 2022; Kildahl et al., 2020b; Rumball et al., 2020) may have affected the participants, and the prevalence of any traumatic experience is likely to have been higher than the current estimate for violence and sexual abuse. Finally, the prevalence of violence or sexual abuse may have been underestimated, as there may have been instances of such events that were not known or reported (e.g. Kildahl, Helverschou, & Oddli, 2020).

## Conclusion

Previous experiences involving violence or sexual abuse were common in a clinical sample of autistic adults with intellectual disabilities, highlighting the importance of trauma screening and assessment in this population. Few participants were diagnosed with PTSD, suggesting that PTSD remains an underutilised diagnosis in this population, and that emotional and behavioural changes associated with traumatic experience are unlikely to be reflected in mental health diagnoses for these individuals. Several potential mechanisms for this lack of recognition are possible, including overlap in the behavioural manifestations for PTSD and anxiety/depressive disorders, failure to explore the meaning of trauma for these individuals, diagnosing PTSD requiring more extensive inferences than diagnosing anxiety/depression in individuals with limited verbal communication skills, and clinicians having become accustomed to stress/anxiety/trauma in autistic individuals and therefore view these as intrinsic to autism. Finally, identified associations between violence, sexual abuse and a measure of ‘challenging’ behaviours provide further support of the previously identified link between trauma/PTSD symptoms and ‘challenging’ behaviours.
